# NF-κB activation is an early event of changes in gene regulation for acquiring drug resistance in human adenocarcinoma PC-9 cells

**DOI:** 10.1371/journal.pone.0201796

**Published:** 2018-08-03

**Authors:** Masashi Fukuoka, Katsuji Yoshioka, Hirohiko Hohjoh

**Affiliations:** 1 Department of Molecular Pharmacology, National Institute of Neuroscience, NCNP, Kodaira, Tokyo, Japan; 2 Division of Molecular Cell Signaling, Cancer Research Institute, Kanazawa University, Kanazawa, Japan; University of South Alabama Mitchell Cancer Institute, UNITED STATES

## Abstract

Gefitinib and erlotinib are epidermal growth factor receptor-tyrosine kinase inhibitors (EGFR-TKIs). Although EGFR-TKIs are effective as anti-cancer drugs, cancer cells sometimes gain tolerance to the drugs. Previous studies suggested that the fibroblast growth factor receptor (FGFR)-signaling pathway could serve as compensation for the EGFR-signaling pathway inhibited by EGFR-TKIs. Our study further suggested that FGF2, a FGFR ligand, leaked out from naïve cells killed by gefitinib could initiate the FGFR-signaling pathway in surviving cells; *i*.*e*., altruistic survival may occur in naïve cells immediately after EGFR-TKI treatment. Altruistic survival may be temporal, and cells need to change their gene regulation toward gaining resistance to EGFR-TKIs. Changes in such gene regulation after EGFR-TKI treatment are poorly understood. In this study, we examined early events of such gene regulation changes in human adenocarcinoma PC-9 cells that are capable of changing their nature from susceptibility to resistance to EFGR-TKIs. Our study indicated that activation of nuclear factor-kappa B (NF-κB) occurred in the cells immediately after EGFR-TKI treatment and also by gene silencing against oncogenic *EGFR*; and, MG132 treatment for inhibiting NF-κB activation affected cell viability. Taken together, our findings (including the previous study) suggest that altruistic survival and NF-κB activation might be vital for initiating the acquisition of EGFR-TKI resistance.

## Introduction

Epidermal growth factor receptor (EGFR) is a transmembrane protein containing a tyrosine kinase domain in its intracellular region. Binding of epidermal growth factor (EGF) to EGFR triggers the dimerization of EGFR followed by autophosphorylation of its tyrosine residues, thereby activating its downstream signaling-pathway involving ERK/MAPK and PI3K-AKT-mTOR[1,2]. The EGFR-signaling system plays important roles in various vital functions such as cell proliferation and cell survival.

*EGFR* mutations have been found in various cancers, and some of the mutations may confer continuous activation of EGFR[2–5]. To suppress such abnormal activation of EGFR, EGFR-tyrosine kinase inhibitors (EGFR-TKIs), *e*.*g*., gefitinib and erlotinib, have been developed and used as anti-cancer drugs[4–6]. Although EGFR-TKI is an effective therapeutic agent, a long-term treatment with EGFR-TKI sometimes leads cancer cells into EGFR-TKI-tolerance[4,7,8], by which therapeutic options are probably reduced in cancer patients. To understand the mechanism of such drug resistance and to avoid the acquisition of drug tolerance, a number of studies have been made, and genetic variations capable of conferring drug resistance have been identified in genes that are directly or indirectly associated with drugs used[9–16].

Our previous study[8] focused on the early stages of EGFR-TKI-treatment and examined how naïve cells were able to survive from the first exposure of EGFR-TKIs before the occurrence of favorable genetic variations for resistance of the drugs. Our findings suggested that the fibroblast growth factor receptor (FGFR) and its ligand FGF2 could work as a surrogate signaling pathway under the inhibition of the EGFR-signaling pathway by EGFR-TKIs. Further interestingly, FGF2 that can initiate the FGFR-signaling pathway appeared to be supplied from neighboring cells killed by EGFR-TKIs. Thus, altruistic survival may occur in naïve cells that exposed to EGFR-TKIs for the first time.

Although naïve cells can escape from the first exposure of EGFR-TKIs by temporal altruistic survival, the cells could hardly keep on living unless they could change their gene regulation to acquire drug resistance. Our current study focused on the initial events of gene regulation change in human adenocarcinoma PC-9 cells. Such early changes as well as altruistic survival might be vital for acquiring EGFR-TKI resistance in PC-9 cells.

## Materials and methods

All the methods in this study were carried out in accordance with the guidelines of National Institute of Neuroscience, National Center of Neurology and Psychiatry (NCNP), Japan. All the genetic recombination experiments in this study were approved by the committee for safety recombinant DNA experiment in National Institute of Neuroscience, NCNP Japan.

### Reagents

Chemicals used in this study were as follows: gefitinib (JS Research Chemicals Trading e. Kfm., Schleswig Holstein, Germany), erlotinib (Cayman Chemical Company, Ann Arbor, MI, USA), PD173074 (Merck Millipore, Billerica, MA, USA), MG132 (Cell signaling technology, Danvers, MA, USA) and FGF2 (ReproCELL Incorporated, Yokohama, Japan). The agents except for FGF2 were dissolved in dimethyl sulfoxide (DMSO) (Sigma-Aldrich, St Louis, MO, USA). For neutralization of extracellular FGF2 in culture medium, anti-FGF2 antibody (#05–117, clone bFM-1; Merck Millipore) was used.

### Cell culture

PC-9 cells, a human non-small cell lung cancer cell line, were obtained from Immuno-Biological Laboratories (No.37012), and grown in RPMI1640 medium (Wako, Osaka, Japan) supplemented with 10% fetal bovine serum (Thermo Fisher Scientific, Waltham, MA, USA), 100 units/ml penicillin, and 100 μg/ml streptomycin (Wako) at 37°C in a 5% CO_2_ humidified chamber.

### Transfection of reporter plasmids and reporter assay

The day before transfection, cells were trypsinized, diluted with medium without antibiotics, and seeded onto 96-well culture plates (0.5x10^4^ cells/well). The pGL4 vectors carrying various response elements followed by the *Photinus luciferase* reporter gene (40ng/well) (Promega, Fitchburg, WI, USA) were introduced together with the phRL-TK plasmid (10ng/well) (Promega) into cells by using a Lipofectamine2000 transfection reagent (Thermo Fisher Scientific) according to the manufacturer’s instructions. After 24h-incubation, culture media were replaced with fresh medium containing 10μM of gefitinib or erlotinib, and further incubation was carried out for 6h. After the treatment, cells were lysed, and the expression levels of the *Photinus* and *Renilla luciferase* reporter genes were examined by a Dual-Luciferase Reporter Assay system (Promega) according to the manufacturer’s instructions. The luminescent signals were measured using a Synergy H1 Multi-Mode Reader (BioTek, Winooski, VT, USA).

The pGL4 vectors used in this study were as follows (abbreviated name used in this study):

pGL4-27[*luc2P*/minP/Hygro](minP),
pGL4.29[*luc2P*/CRE/Hygro](CRE),
pGL4.30[luc2P/NFAT-RE/Hygro](NFAT-RE),
pGL4.32[*luc2P*/NF-κB-RE/Hygro](NF-κB-RE),
pGL4.33[*luc2P*/SRE/Hygro](SRE),
pGL4.34[*luc2P*/SRF-RE/Hygro](SRF-RE),
pGL4.37[*luc2P*/ARE/Hygro](ARE),
pGL4.38[luc2P/p53 RE/Hygro](p53 RE),
pGL4.39[luc2P/ATF6 RE/Hygro](ATF RE),
pGL4.40[luc2P/MRE/Hygro](MRE),
pGL4-41[*luc2P*/HSE/Hygro](HSE),
pGL4.42[luc2P/HRE/Hygro](HRE),
pGL4.43[luc2P/XRE/Hygro](XRE),
pGL4.44[luc2P/AP1 RE/Hygro](AP1 RE),
pGL4.45[luc2P/ISRE/Hygro](ISRE),
pGL4.47[luc2P/SIE/Hygro](SIE),
pGL4.48[luc2P/SBE/Hygro](SBE),
pGL4.49[luc2P/TCF-LEF RE/Hygro](TCF-LEF RE),
pGL4.52[*luc2P*/STAT5 RE/Hygro](STAT5-RE).

### Gene silencing by RNAi

To knockdown *EGFR*, a small interfering RNA (siRNA) against the gene as a whole was purchased from Thermo Fisher Scientific (catalog-number: s564). In addition, si746/50_3D10 was used for oncogenic allele-specific silencing against a deletion-type of *EGFR* in PC-9 cells according to the previous study[17]. The sequences of si746/50_3D10 are as follows:

5’- CGCUAUCAAAACAUCUCCGUU-3’

5’- CGGAGAUGUUUUGAUAGCGUU-3’

The siRNAs (10-20nM final concentration) were transfected into cells by a Lipofectamine2000 transfection reagent (Thermo Fisher Scientific) according to the manufacturer’s instructions. The cells were incubated for 24h and subjected to subsequent experiments.

### Total RNA preparation and reverse transcription (RT)-quantitative polymerase chain reaction (qPCR)

Total RNAs were extracted from cells using a TRI Reagent (MRC, Cincinnati, OH, USA) according to the manufacturer’s instructions, and subjected to complementary DNA (cDNA) synthesis using oligo (dT)_15_ primers (Promega) and a Superscript III reverse transcriptase (Thermo Fisher Scientific) according to the manufacturer’s instructions. The resultant cDNAs were subjected to qPCR using a StepOne Plus Real-Time PCR system (Thermo Fisher Scientific) with a FastStart Universal SYBR Green Master (Roche Diagnostics, Basel, Switzerland) and Perfect Real Time primers (TAKARA BIO, Kusatsu, shiga, Japan) according to the manufacturers’ instructions. The Perfect Real Time primers used were as follows (TAKARA BIO primer-set IDs):

*EGFR* (HA159668), *GAPDH* (HA067812), *GFOD1* (HA205357).

### Western blot analysis

Cells were washed with D-PBS (Wako) and lysed in RIPA buffer (Thermo Fisher Scientific) containing 1x Protease/Phosphatase Inhibitor Cocktail (Cell signaling technology). The lysate was incubated on ice for 5min, passed 10 times through a 26G needle using a 1ml-syringe and centrifuged at 14,000xg for 15min at 4°C. The resultant supernatant (cell lysate) was collected. Protein concentration of the cell lysate was measured by a protein quantification kit-wide range (DOJINDO, Mashiki-town, Kumamoto, Japan). Equal amounts of protein (≈40μg) were mixed with 2x sample buffer (125mM Tris-HCl pH6.8, 2% glycerol, 4% SDS, 0.02% bromophenol blue, 10% beta-mercaptoethanol) and boiled for 5min. The protein samples were electrophoretically separated on 10% SDS-polyacrylamide gels (SDS-PAGE), and blotted onto polyvinylidene fluoride membranes (Immobilon P; Merck Millipore). The membranes were incubated for 1h in blocking buffer (TBS-T containing 5% skim milk) and then with diluted primary antibodies at 4°C overnight or at room temperature for 1h. After incubation, the membranes were washed in TBS-T, and incubated with 1/5,000 diluted horseradish peroxidase-conjugated goat anti-mouse IgG (Sigma-Aldrich) or goat anti-rabbit IgG (Sigma-Aldrich) for 30min at room temperature. Antigen-antibody complexes were visualized using an ECL Prime Western Blotting Detection Reagent (Merck Millipore) according to the manufacturer’s instructions. The primary antibodies used in Western blotting and their product IDs and dilution ratios in parentheses were as follows:

Anti-EGFR (#2232; 1/1,000), anti-IκBα (#4814; 1/1,000), phosphor IκBα (#9246; 1/500) and anti-GAPDH (#2118; 1:2000) were purchased from Cell Signaling Technology. Anti-α-Tubulin (F2168; 1/5,000) were purchased from Sigma-Aldrich. Anti-FGF2 (#05–118; 1/1000) were purchased from Merck Millipore.

### Cell viability assay

Cell viability was measured by a CellTiter 96^®^ Aqueous Non-Radioactive Cell Proliferation Assay (Promega) according to the manufacturer’s instructions.

### ELISA analysis

Conditioned media from PC-9 cells were collected and centrifuged at 2,000xg for 15min at room temperature. The supernatant was transferred into an Amicon^®^ Ultra centrifugal filter 10k (Merck Millipore), and subjected to concentration by centrifugation at 14,000xg for 15min. The level of FGF2 in the concentrated medium was measured by a Human FGF basic Quantikine ELISA kit (R&D SYSTEMS, Minneapolis, MN, USA) according to the manufacturer’s instructions.

### Prediction of transcription factor binding sites

Transcription factor binding sites in a putative promoter region of the *GFOD1* gene was predicted by using the TFBIND software according to the instructions[18].

## Results

### Reporter assay for activated transcription factors in PC-9 cells after EGFR-TKI treatment

**Fig 1:**
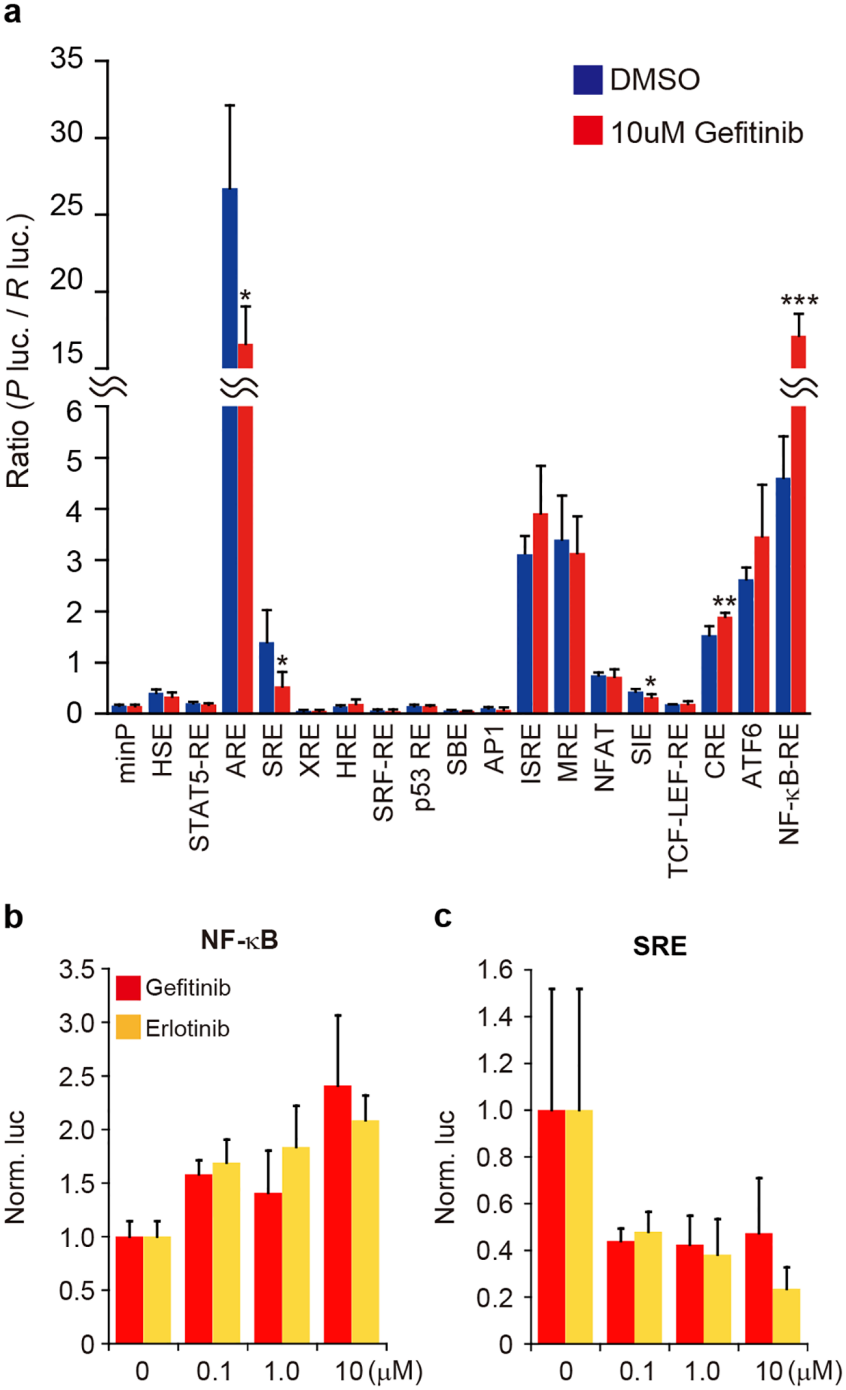
Reporter gene assay for transcription factors influenced by EGFR-TKIs. (**a**) Luciferase reporter assay. The pGL4 vectors, which encode response elements for transcription factors (indicated) or a minimal promoter (minP; a negative control) linked with the *Photinus luciferase* gene, were transfected together with phRL-TK carrying the *Renilla luciferase* gene (as a reference) into PC-9 cells. After 24h-incubation, the cells were exposed to 10μM gefitinib or DMSO (a vehicle) for 6h and subjected to a dual-luciferase assay. The activity of the *Photinus* luciferase (*P* luc.) was normalized to that of the *Renilla* luciferase (*R* luc.). Data are shown as mean ± standard deviation (SD) (*n* = 4). Significant difference between gefitinib and DMSO was examined by means of two-tailed Student’s t-test (*<0.05, **p<0.01, ***p<0.001). (**b**, **c**) Effects of gefitinib or erlotinib on transcription factors. The GL4-plasmid carrying the NF-κB response element (**b**) or the serum response element (SRE) (**c**) was co-transfected with phRL-TK into PC-9 cells, and after 24h-incubation, the cells were treated with 0, 0.1, 1.0 and 10μM of gefitinib or erlotinib for 6h. Dual-luciferase assay followed by normalization were carried out as in **a**. Normalized data were further normalized to the data obtained from cells treated with no EGFR-TKIs (0 μM) as 1. Data are shown as mean ± standard deviation (SD) (*n* = 3).

When naïve cells acquire tolerance under the presence of harmful drugs, gene expression changes should occur in the cells. Identification of such gene expression changes immediately after drug treatment is of particular importance, but not well performed. To explore such a change in gene regulation, human adenocarcinoma PC-9 cells may be a good cellular model: this is because the cells are capable of changing their nature from susceptibility to resistance to EFGR-TKIs[8,9]. To search for early events of gene regulation immediately after EGFR-TKIs treatment, we focused on transcription factors and examined the effects of EGFR-TKIs on transcription factors by means of a reporter-gene assay. We used the pGL4 reporter plasmids carrying various transcription factor response elements linked to the *luciferase* reporter gene, and transfected them into naïve PC-9 cells. The expression of the reporter gene was examined after the cells were treated with gefitinib, an EGFR-TKI, for 6h. As a result, the expression of the reporter gene carrying the response element of nuclear factor-kappa B (NF-κB) was significantly upregulated under the presence of gefitinib (Fig 1a). A slight increase in the expression of the reporter gene carrying the cAMP response element (CRE) was also detected after gefitinib treatment. When the reporter genes carrying the antioxidant response element (ARE), serum response element (SRE) and sis-inducible element (SIE) were examined in the presence of gefitinib, a significant decrease in the reporter gene expression was seen. Note that not only gefitinib but also erlotinib had an effect on the NF-κB response element and the serum response element (SRE) (Fig 1b and 1c); and the dose-dependent effect of erlotinib on NF-κB activation was also detected (Fig 1b). Consequently, gefitinib and erlotinib exhibited similar results in the reporter gene assay.

### Effects of extracellular FGF2 on cell survival and on activation of transcription factors

**Fig 2:**
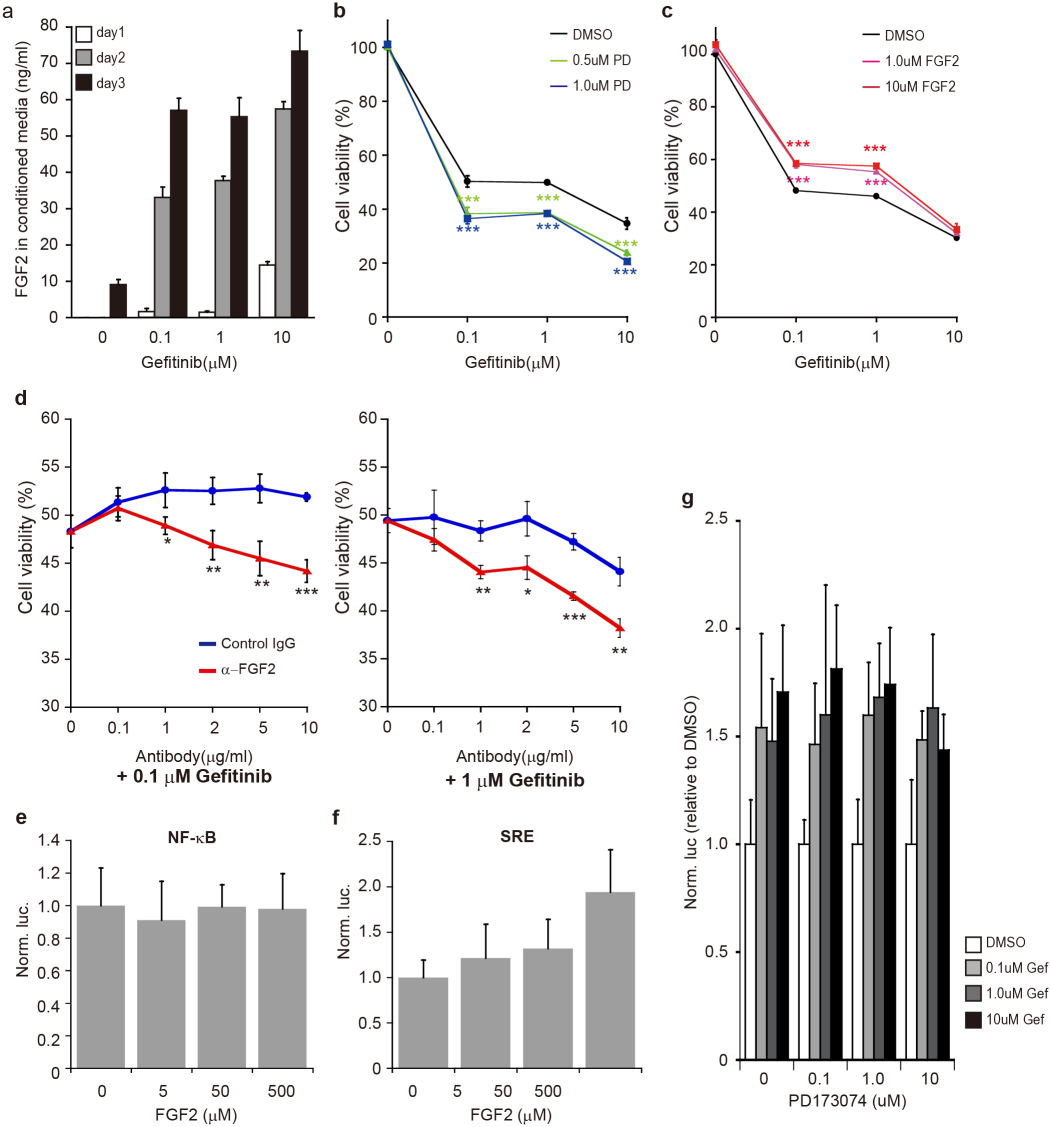
Effects of extracellular FGF2 on cell survival and transcriptional activation. (a) ELISA. PC-9 cells were treated with various concentrations of gefitinib (indicated) for indicated days, and conditioned media were collected. The FGF2 level in the media was examined by an ELISA analysis. Data are shown as mean ± SD (*n* = 3). (b) Effects of PD173074 (PD), an FGFR inhibitor, on cell viability under the presence of gefitinib. PC-9 cells were treated with 0.5 and 1.0μM of PD173074 or DMSO (a vehicle) for 1h prior to exposure to 0, 0.1, 1.0 and 10μM of gefitinib. After 3day-incubation, cell viability was examined. Data are shown as mean ± SD (*n* = 3). The data of DMSO represent the results treated only by gefitinib. Significant difference between PD173074 and DMSO, *i*.*e*., between the presence and absence of PD173074, was examined by one-way analysis of variance (ANOVA) (Dunnett’s test; ***p<0.001). (c) Effects of extracellular FGF2 on cell viability under the presence of gefitinib. PC-9 cells were treated with FGF2 or DMSO together with gefitinib, and cell viability was examined and analyzed as in b (*n* = 3). (d) Inhibition of extracellular FGF2 by neutralizing antibodies. PC-9 cells were treated with 0.1 or 1μM of gefitinib under the presence of various levels of anti-FGF2 antibodies (α-FGF2). After 3day-incubation, cell viability was examined as in b. Significant difference between α-FGF2 and a control IgG was examined by Student’s t-test (two-tailed; *<0.05, **p<0.01, ***p<0.001). (e, f) Effects of extracellular FGF2 on transcription factors. The pGL4-NF-κB-RE and pGL4-SRE plasmids were introduced together with phRL-Tk into PC-9 cells. After 24h-incubation, the cells were treated with various levels of FGF2, and further incubation was carried out. After 6h, dual-luciferase assay and data analyses were carried out as in Fig 1b (*n* = 3). (g) Influence of PD173074 on NF-κB activation. The pGL4-NF-κB-RE and phRL-Tk plasmids were transfected into PC9 cells as in e, and the cells were treated with PD173074 for 1h prior to gefitinib treatment. After 6h gefitinib treatment, dual-luciferase assay was carried out as in Fig 1b (*n* = 3).

The previous study suggested that FGF2, a ligand of FGFR, could leak out from neighboring cells killed by gefitinib and could trigger the initiation of the FGFR-signaling pathway compensating the EGFR-signaling pathway inhibited by gefitinib[8]. Consistently, the gefitinib-dose-dependent increase of extracellular FGF2 that leaked from gefitinib-treated cells (Fig 2a), and also the positive contribution of FGF2/FGFR to cell survival (Fig 2b and 2c) were reproducibly observed in this study. In addition, little or no increase in endogenous FGF2 was detected in gefitinib-treated cells (S1 Fig), suggesting that the increase in extracellular FGF2 after gefitinib treatment was not caused by increasing endogenous FGF2. To further prove the vital effect of leaked-out (dead-cell-derived) FGF2, PC-9 cells were treated with gefitinib together with anti-FGF2 antibodies for neutralizing extracellular FGF2. The results indicated that cell viability was significantly reduced with increasing in the dose of anti-FGF2 antibodies (Fig 2d); thus, the neutralization of leaked-out FGF2 resulted in worsening of cell viability. Therefore, the findings strongly suggested that extracellular FGF2 leaked out from dead cells was essential to survive cells which were still alive under the presence of gefitinib.

Based on the positive contribution of FGF2 to cell survival, we examined whether extracellular FGF2 was capable of activating NF-κB in the absence of gefitinib. PC-9 cells that were transfected with the reporter gene carrying the NF-κB response element (NF-κB-RE) were treated with various concentrations of FGF2, and the expression of the reporter gene was examined. As a result, no significant difference in the reporter gene expression among the various doses of FGF2 was detected (Fig 2e). When the reporter gene carrying SRE was examined, an increased tendency of the expression with increasing in FGF2 was observed (Fig 2f), suggesting SRE reactive to FGF2. To further confirm, we examined the effect of gefitinib on NF-κB activation under the presence of PD173074, a FGFR-tyrosine kinase inhibitor. The results indicated that the activation of NF-κB was hardly affected by FGFR inhibition (Fig 2g). Therefore, the findings suggested that neither FGF2 nor FGFR-signaling pathway was implicated in NF-κB activation under the presence of gefitinib.

### Effects of *EGFR* knockdown on activation of NF-κB

The contribution of gefitinib to the activation of NF-κB was obvious from the current findings described above. However, it was still unclear whether gefitinib triggered the activation of NF-κB through loss of EGFR function or else by unknown function(s) of gefitinib itself. To address the question, we conducted gene silencing against *EGFR* by RNA interference (RNAi). Because PC-9 cells carry oncogenic *E746_A750del EGFR* and normal *EGFR* alleles, we performed RNAi specific for the oncogenic *EGFR* allele as well as for all *EGFR* alleles (including the oncogenic *EGFR* allele). After RNAi knockdown, the reporter gene carrying NF-κB-RE was introduced. If functional knockdown of EGFR were related to the activation of NF-κB, gene silencing of *EGFR* should result similar to that obtained from gefitinib treatment. If gefitinib itself had unknown functions involved in NF-κB activation, RNAi knockdown could lead to little activation of NF-κB.

**Fig 3:**
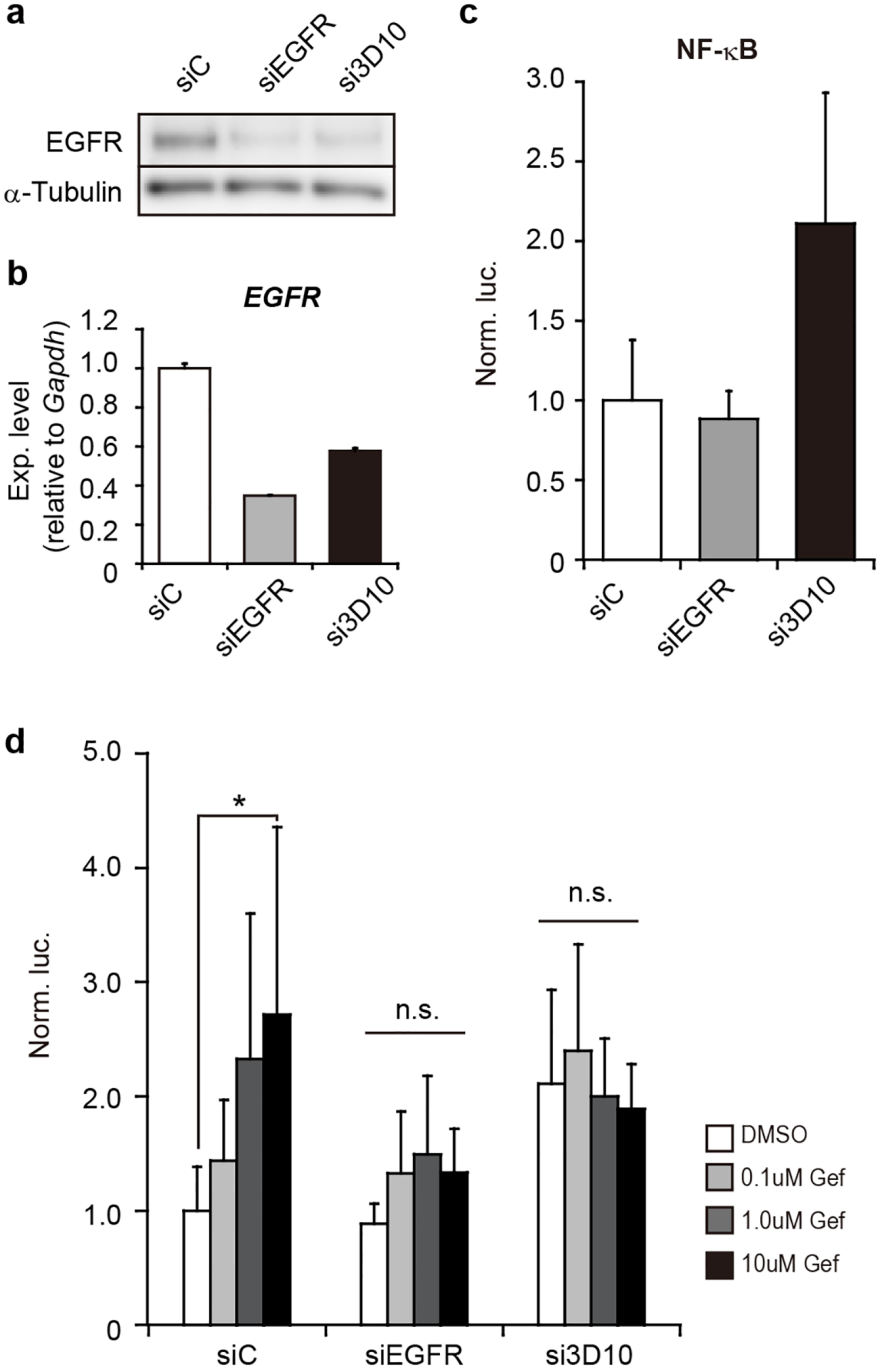
Effect of gene silencing against *EGFR* on NF-κB activation. (**a**) RNAi knockdown. PC-9 cells were subjected to gene silencing with siRNAs against total *EGFR* (siEGFR) or oncogenic mutant *EGFR* alleles [si746/50_3D10 (si3D10) targeting the 746/750 deletion in exon 19] in PC-9 cells. Non-silencing siRNAs (siControl: siC) were also examined as a negative control. 24h after introduction of siRNAs, EGFR was examined by western blotting. Alpha-tubulin was examined as an internal control. (**b**) *EGFR* mRNA levels. Indicated siRNAs were transfected into PC-9 cells as in **a**. The level of *EGFR* was examined by RT-qPCR and analyzed by the delta-delta Ct method using the data of *Gapdh* as a reference. The data were further normalized to the data obtained by siControl (siC) as 1. Data are shown as mean ± SD (*n* = 3). (**c**) Activation of NF-κB. The pGL4-NF-κB-RE and phRL-Tk plasmids together with indicated siRNAs (20nM) were introduced into PC-9 cells. After 24h incubation, Dual-luciferase assay was carried out as in Fig 1b (*n* = 6). (**d**) Effect of gefitinib on NF-κB activation under *EGFR* knockdown. Reporter plasmids and siRNAs were introduced into PC9 cells as in **c**. The cells were treated with 0, 0.1, 1.0 and 10μM of gefitinib (Gef). Dual-luciferase assay was carried out as in Fig 1b (*n* = 6). Significant difference in cell viability between gefitinib and DMSO treatment was examined by one-way analysis of variance (ANOVA) (Dunnett’s test; *p<0.05). n.s.: not significant.

As shown in Fig 3c, the results indicated that the reporter gene was upregulated under knockdown of the oncogenic *EGFR* (si3D10). Interestingly, total *EGFR* knockdown exhibited no upregulation of the reporter gene (siEGFR in Fig 3c), and gefitinib treatment under such total *EGFR* knockdown had little effect on the expression of the reporter gene (Fig 3d); thus, suggesting little possibility of gefitinib’s own unknown function. Taken together, the findings suggested that the loss of oncogenic EGFR function might be involved in the activation of NF-κB.

### Cell viability under inhibition of NF-κB activation

Inhibition of NF-κB might have some influence on cell survival if activated NF-κB had some relationship with the survival of PC-9 cells under the presence of EGFR-TKIs. We investigated if the cell viability of PC-9 cells was affected by inhibiting NF-κB activation under the presence of gefitinib. NF-κB generally forms a protein-complex together with one of the Rel proteins, and the complex was inactivated and sequestered in the cytoplasm by binding of Inhibitory κB (IκB). When cells receive stimuli, *e*.*g*., a proinflammatory cytokine such as tumor necrosis factor-alpha (TNFα), the stimulation triggers the activation of IκB kinases (IKKs) followed by phosphorylation of IκB. The phosphorylated IκB becomes a target of ubiquitination and/or proteasomal degradation, thereby freeing the NF-κB complex as an active form from IκB; and then, the NF-κB complex can move into the nucleus and work as a transcription factor (refer to review articles[19–22]).

**Fig 4:**
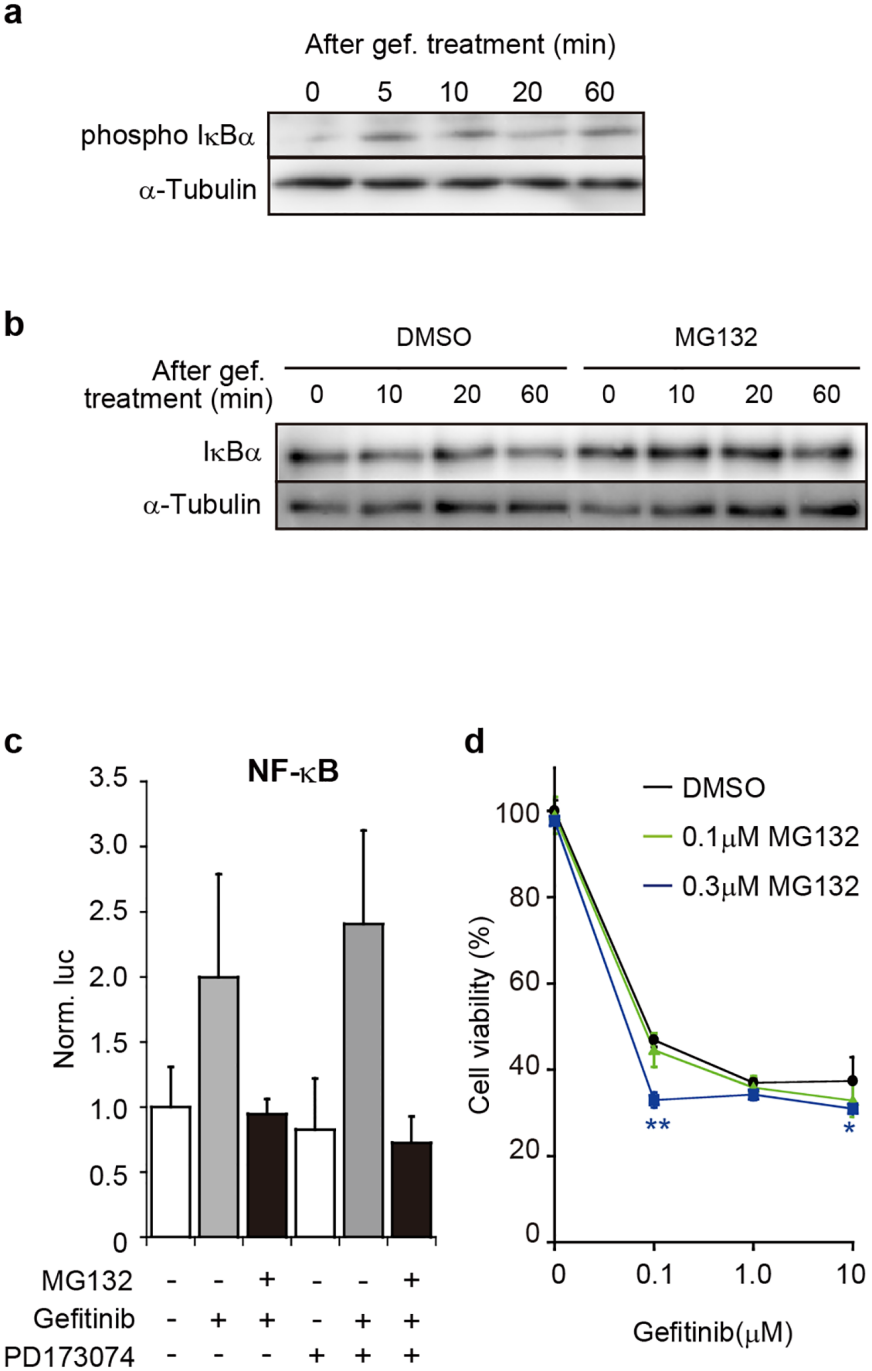
Effects of MG132 on gefitinib-induced NF-κB activation and cell viability. (**a**) Phosphorylation of IκBα after gefitinib treatment. PC-9 cells were treated with 80μM of gefitinib (gef.), and cell lysates were prepared at indicated times. Phosphorylated IκBα (phospho IκBα) and α-Tubulin as an internal control were examined by western blotting. (**b**) Inhibition of IκBα degradation by MG132. PC-9 cells were treated with 10μM of MG132 or DMSO (vehicle) for 1h prior to 10μM gefitinib treatment. Total IκBα and α-Tubulin were examined by western blotting. (**c**) Reporter gene assay. The pGL4-NF-κB-RE and phRL-Tk plasmids were transfected into PC-9 cells. After 24h-incubation, the cells were treated with 1μM of MG132 or DMSO for 1h and then treated with 10μM of gefitinib and/or 1.0μM of PD173074 for 6h. The expression of reporter genes was examined and analyzed as in Fig 1b. Data are shown as mean ± SD (*n* = 4). (**d**) Cell viability. PC-9 cells were treated with MG132 or DMSO (as a control) for 1h and then treated with 0, 0.1, 1.0 and 10μM of gefitinib. After 3days of gefitinib treatment, cell viability was examined. Data are shown as mean ± SD (*n* = 3). Significant differences in cell viability between MG132 and DMSO treatment were analyzed by one-way analysis of variance (ANOVA) (Dunnett’s test; *p<0.05, **p<0.01).

The phosphorylation of IκB immediately after gefitinib treatment was detected (Fig 4a). Thus, the result suggested that a common process of activation of NF-κB occurred in PC-9 cells after gefitinib treatment. To block the activation process, we used MG132, a proteasome inhibitor capable of inhibiting the degradation of IκB. The inhibitory effect of MG132 on IκB degradation was consistently detected (Fig 4b); and, MG132 treatment caused reduction of the expression of the reporter gene carrying NF-κB-RE even in the presence of gefitinib (Fig 4c), indicating that NF-κB activation due to gefitinib was suppressed by MG132. As additional data, neither the presence nor absence of PD173074 had influence in the results, suggesting little contribution of FGFR to NF-κB activation.

When PC-9 cells were treated with gefitinib and MG132, in other words, when the cells were treated with gefitinib without NF-κB-activation, a significant decrease in cell viability was detected at a lower concentration of gefitinib (0.1μM) under the presence of 0.3μM of MG132 (Fig 4d). Therefore, the findings suggested that NF-κB activation immediately after EGFR-TKIs treatment might be implicated in cell survival.

## Discussion

Drug resistance is a major problem in chemotherapy, and eventually reduces therapeutic options to patients. To solve the problem, elucidation of the mechanism by which cells can acquire drug resistance is vital; and intracellular changes immediately after drug treatment are of particular importance. PC-9 cells examined in this study are capable of developing resistance to EGFR-TKIs by exposure of the drugs[8,9]. Thus, PC-9 cells may be a useful cellular model for examining the mechanism of acquired drug resistance and provide us with the opportunity to investigate intracellular changes in the early stages of EGFR-TKIs treatment.

Previous studies suggested that the FGFR-signaling pathway was capable of working as a surrogate pathway for the EGFR-signaling pathway inhibited by EGFR-TKIs[8,15,16,23], and our study further suggested that FGF2, a key ligand that triggers the initiation of the surrogate FGFR-signaling pathway, would be supplied from neighboring cells killed by EGFR-TKIs[8]. Thus, altruistic survival based on neighbors’ self-sacrifices may have an important role in cell survival early in the treatment of EGFR-TKIs (Fig 2).

Even if cells could escape from the first exposure of EGFR-TKIs by temporal altruistic survival, the cells would be killed by the drugs unless they could develop resistance to the drugs. Therefore, surviving cells need to change their properties to be resistant to EGFR-TKIs as soon as possible; and of such changes, changes in gene regulation would be vital.

Our current study indicated NF-κB activation immediately after EGFR-TKIs treatment as an initial event of such changes in PC-9 cells (Fig 1). The activation of NF-κB was independent of the FGFR-signaling pathway, which works as a surrogate pathway for the EGFR-signaling pathway inhibited by EGFR-TKIs (Fig 2). The arrest of EGFR-tyrosine kinase activity appeared to be a key step in the activation of NF-κB.

PC-9 cells possess oncogenic E746_A750del EGFR and normal EGFR, and the oncogenic EGFR promotes tyrosine kinase activity. RNAi knockdown specific for the oncogenic *EGFR* resulted in the activation of NF-κB (Fig 3). Since the oncogenic E746_A750del EGFR is the major target of EGFR-TKIs, inhibition of oncogenic EGFR function might trigger the onset of NF-κB activation. The interesting point to note here is that total EGFR knockdown by RNAi exhibited little activation of NF-κB even under the presence of gefitinib (Fig 3c and 3d). The finding led us to the possibility that normal EGFR might have some role in the activation of NF-κB after inhibition of oncogenic EGFR. To clarify the possibility, further studies need to be carried out in the future.

It is of importance to find evidence on an association between NF-κB activation and cell survival (viability) under the presence of EGFR-TKIs. MG132 is a proteasome inhibitor that blocks the degradation of IκB binding to NF-κB and consequently inhibits NF-κB activation. When cells were treated with MG132 together with gefitinib, the cells exhibited a decrease in cell viability (Fig 4). This suggests that NF-κB activation might be implicated in cell survival under the presence of EGFR-TKIs. In addition, the findings were consistent with the previous study[13] that identified many genes working in NF-κB-activating cascades as genes associated with resistance to EGFR-TKI. Taken together, activation of NF-κB might participate in early steps to develop resistance to EGFR-TKIs, and might trigger genetic changes for acquiring drug resistance.

NF-κB is a transcription factor and is involved in various gene regulations. Likewise, NF-κB that is activated by EGFR-TKIs presumably participates in gene regulation. As an instance, the expression of the *Glucose-fructose oxidoreductase domain containing 1* (*GFOD1*) gene, which has many predicted NF-κB binding sites in its putative promoter region (S1 Table), was markedly upregulated 24h after gefitinib treatment (S2 Fig) and also increased in gefitinib-resistant PC-9 cells relative to naïve cells[8]. Thus, activated NF-κB may contribute to alteration of gene expression during early stages of EGFR-TKIs treatment and over the course of development of EGFR-TKI resistance.

**Fig 5:**
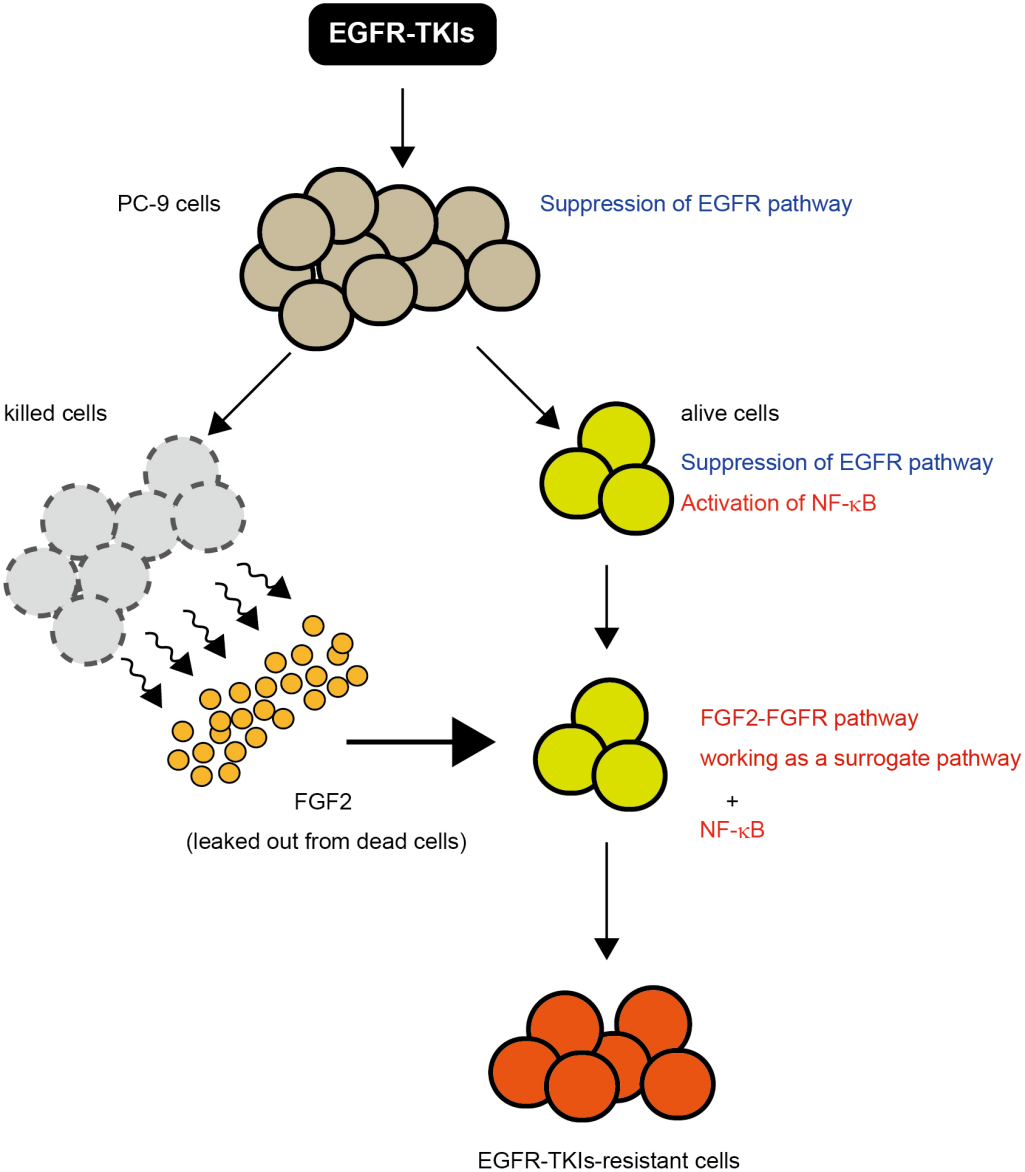
Schematic drawing of hypothetical early events in EGFR-TKIs-exposed PC-9 cells. After EGFR-TKIs treatment, many PC-9 cells are killed and the dead cells leak out FGF2 that works as a survival factor; and simultaneously, neighboring (alive) cells manage to survive from the first exposure of EGFR-TKIs by leaked-out FGF2 and also by NF-kB activation. FGF2 can trigger the initiation of the FGFR-signaling pathway that is a surrogate pathway for suppressed EGFR pathway. The FGFR-signaling pathway and NF-kB independently contribute to cells for survival in the early stages of EGFR-TKIs treatment. Cells that survived successfully, thereafter, need to change gene regulation to maintain resistance to EGFR-TKIs; and NF-kB may play an important role as an initial transcription factor there. Finally, the cells acquire complete drug resistance, in which FGF2-FGFR autocrine system may work as a major vital pathway.

Based on the findings of the previous and present studies concerning the early stages of EGFR-TKIs treatment, a hypothetical scenario may be considered as follows (Fig 5): after EGFR-TKIs treatment, many cells are killed and the cells leak out survival factors such as FGF2; and simultaneously, neighboring (alive) cells may manage to survive from the first exposure of EGFR-TKIs by leaked-out FGF2 and also by activated NF-κB. FGF2 can initiate the FGFR-signaling pathway as a surrogate pathway for the EGFR-signaling pathway inhibited by EGFR-TKIs. NF-κB and FGF2 may independently contribute to cells for survival in the early stages of EGFR-TKIs treatment. Cells which survived successfully, thereafter, change gene regulation to develop and maintain tolerance to EGFR-TKIs; and NF-κB might play an important role as an initial transcription factor there. Finally, the cells would acquire drug resistance, in which FGF2-FGFR autocrine system may work as a major vital pathway[15,16,23]. In conclusion, altruistic survival and NF-κB activation, both of which are induced by EGFR-TKIs treatment, may be essential for cells to survive early in EGFR-TKIs treatment and then to obtain resistance to EGFR-TKIs.

Our current study shed light on the activation of NF-κB immediately after EGFR-TKIs treatment in PC-9 cells capable of acquiring EGFR-TKI resistance. For further investigation, the putative factors mediating the activation of NF-κB under EGFR inhibition still remain unknown. To further clarify the mechanism of acquired EGFR-TKIs resistance, more extensive studies including identification of such factors need to be carried out in the future.

## Supporting information

S1 Fig EGFR and FGF2 under the presence of gefitinib. PC9 cells were treated with 0, 0.1, 10μM of gefitinib. 24h after gefitinib treatment, EGFR and FGF2 were examined by western blotting. GAPDH was examined as an internal control.
(PDF)

S2 Fig *GFOD1* expression after gefitinib treatment. PC-9 cells were treated with gefitinib (indicated) for 24h. The expression level of *GFOD1* was examined by RT-qPCR. The data were analyzed by the delta-delta Ct method using the data of *GAPDH* as an internal reference and with the delta Ct obtained from the data of DMSO as a control as 1. Data are shown as mean +/- SD (n = 3).
(PDF)

S3 Fig Original blot images. Blot images indicated by red boxes were used in Figures.
(PDF)

S1 Table Predicted NF-κB binding sites in a putative *GFOD1* promoter region. NF-κB binding sites in a putative promoter region (1,500bp-long) of *GFOD1* were predicted by the TFBIND software.
(PDF)
