# Supplementary material for: NF-κB activation is an early event of changes in gene regulation for acquiring drug resistance in human adenocarcinoma PC-9 cells
Source: PLoS One. 2018 Aug 3;13(8):e0201796. doi: 10.1371/journal.pone.0201796 (PMC6075786; doi:10.1371/journal.pone.0201796)
Supplement: S1 Fig — PC9 cells were treated with 0, 0.1, 10μM of gefitinib. 24h after gefitinib treatment, EGFR and FGF2 were examined by western blotting. GAPDH was examined as an internal control. (PDF) [file pone.0201796.s001.pdf]

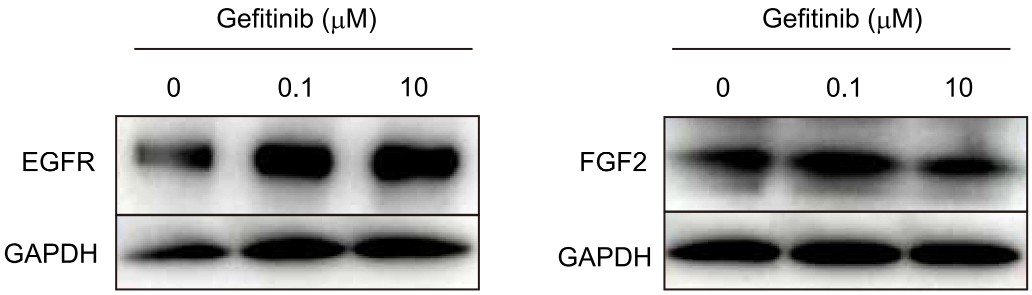

S1 Fig. EGFR and FGF2 under the presence of gefitinib. PC9 cells were treated with 0, 0.1, 10μM of gefitinib. 24h after gefitinib treatment, EGFR and FGF2 were examined by western blotting. GAPDH was examined as an internal control.
