# Supplementary material for: NF-κB activation is an early event of changes in gene regulation for acquiring drug resistance in human adenocarcinoma PC-9 cells
Source: PLoS One. 2018 Aug 3;13(8):e0201796. doi: 10.1371/journal.pone.0201796 (PMC6075786; doi:10.1371/journal.pone.0201796)
Supplement: S2 Fig — PC-9 cells were treated with gefitinib (indicated) for 24h. The expression level of GFOD1 was examined by RT-qPCR. The data were analyzed by the delta-delta Ct method using the data of GAPDH as an internal reference and with the delta Ct obtained from the data of DMSO as a control as 1. Data are shown as mean +/- SD (n = 3). (PDF) [file pone.0201796.s002.pdf]

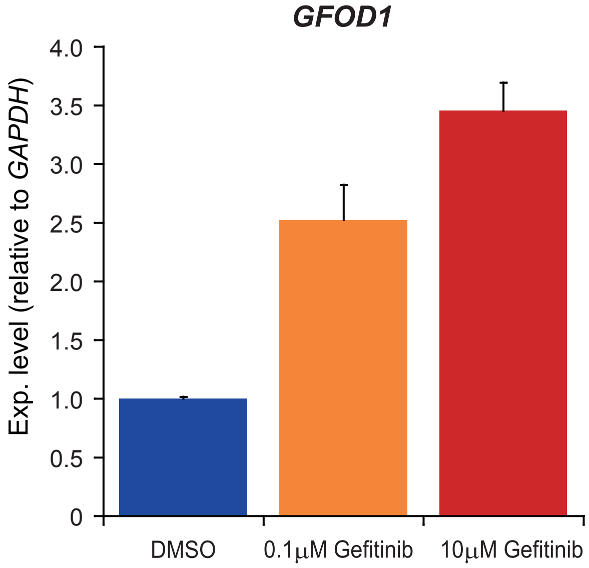

S2 Fig. *GFOD1* expression after gefitinib treatment. PC-9 cells were treated with gefitinib (indicated) for 24h. The expression level of *GFOD1* was examined by RT-qPCR. The data were analyzed by the delta-delta Ct method using the data of *GAPDH* as an internal reference and with the delta Ct obtained from the data of DMSO as a control as 1. Data are shown as mean +/- SD (n = 3 determinations).
