# Supplementary material for: NF-κB activation is an early event of changes in gene regulation for acquiring drug resistance in human adenocarcinoma PC-9 cells
Source: PLoS One. 2018 Aug 3;13(8):e0201796. doi: 10.1371/journal.pone.0201796 (PMC6075786; doi:10.1371/journal.pone.0201796)
Supplement: S3 Fig — Blot images indicated by red boxes were used in Figures. (PDF) [file pone.0201796.s003.pdf]

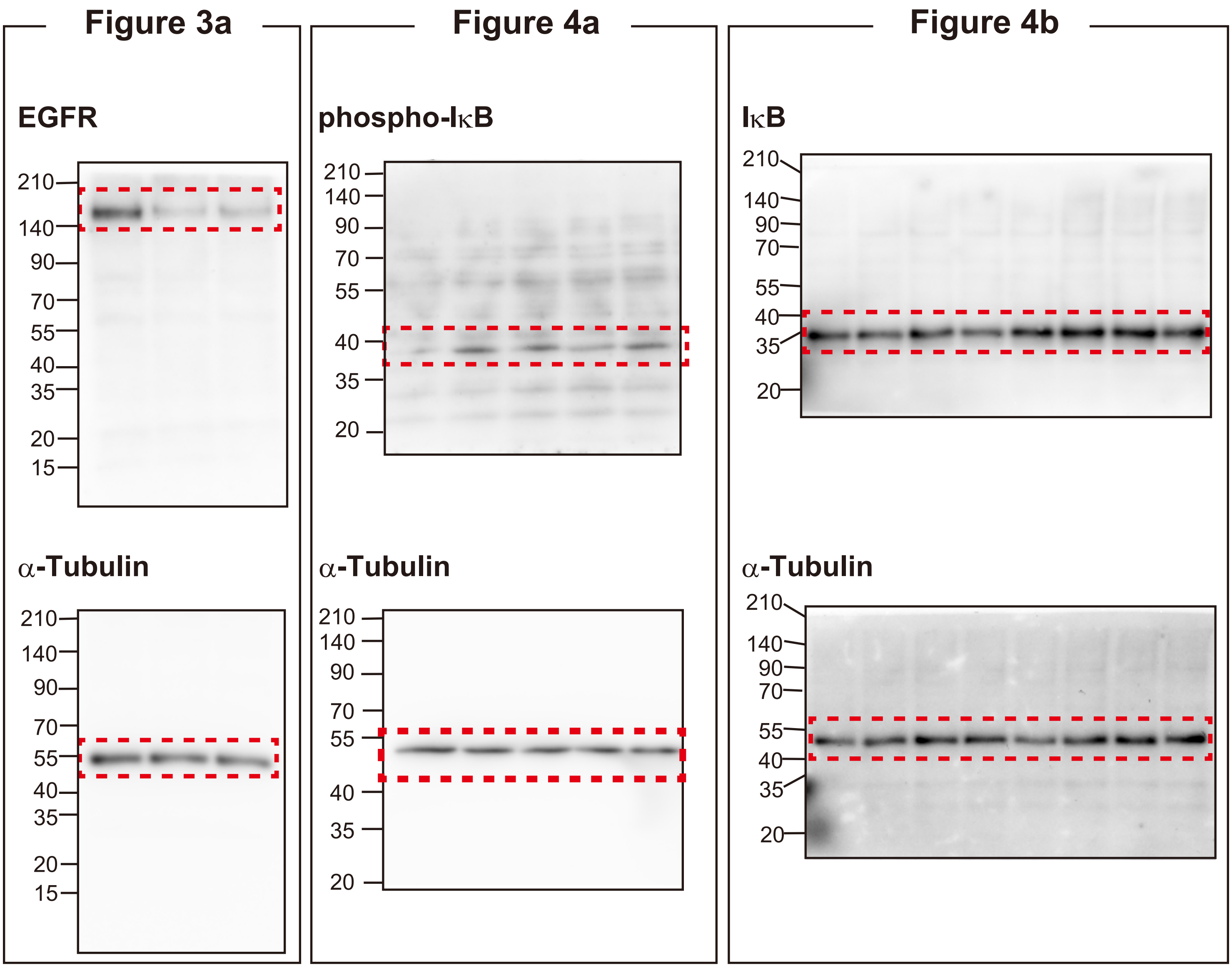

S3 Fig. Original blot images. Blot images indicated by red boxes were used in Figures.
