# Supplementary material for: NF-κB activation is an early event of changes in gene regulation for acquiring drug resistance in human adenocarcinoma PC-9 cells
Source: PLoS One. 2018 Aug 3;13(8):e0201796. doi: 10.1371/journal.pone.0201796 (PMC6075786; doi:10.1371/journal.pone.0201796)
Supplement: S1 Table — NF-κB binding sites in a putative promoter region (1,500bp-long) of GFOD1 were predicted by the TFBIND software. (PDF) [file pone.0201796.s004.pdf]

S1 Table

Transcription factor label	Similarity	Position[a]	Consensus sequence
M00051 V$NFKAPPAB50_01	0.756997	69 (-)	GGGGATYCCC GCGCCGCCCC
M00051 V$NFKAPPAB50_01	0.753665	124 (+)	GGGGATYCCC CGGGAGCCCA
M00051 V$NFKAPPAB50_01	0.772323	124 (-)	GGGGATYCCC CGGGAGCCCA
M00054 V$NFKAPPAB_01	0.78402	443 (+)	GGGAMTTYCC GGTATTGTCC
M00054 V$NFKAPPAB_01	0.817978	444 (-)	GGGAMTTYCC GTATTGTCCC
M00051 V$NFKAPPAB50_01	0.795646	716 (-)	GGGGATYCCC CGGGCTCCCA
M00051 V$NFKAPPAB50_01	0.847845	806 (+)	GGGGATYCCC GGGGGTCTCC
M00051 V$NFKAPPAB50_01	0.790982	806 (-)	GGGGATYCCC GGGGGTCTCC
M00052 V$NFKAPPAB65_01	0.851018	806 (+)	GGGRATTTCC GGGGGTCTCC
M00054 V$NFKAPPAB_01	0.847191	806 (+)	GGGAMTTYCC GGGGGTCTCC
M00051 V$NFKAPPAB50_01	0.784096	807 (+)	GGGGATYCCC GGGGTCTCCG
M00051 V$NFKAPPAB50_01	0.760107	1043 (-)	GGGGATYCCC GGGACTCTGC
M00054 V$NFKAPPAB_01	0.808489	1043 (+)	GGGAMTTYCC GGGACTCTGC

NF-kB binding sites in a putative promoter region (1,500bp-long) of GFOD1 were predicted by the TFBIND software.

a: Position on the putative GFOD1 promoter sequence from position 1 that is equal to the 3’ end of the promoter sequence and which is adjacent to the GFOD1 coding sequence (NM_018988.3).
(+) and (-) means forward and reverse strands, respectively.
